# Inclusive Human Resource Management and Nurses' Innovative Behavior during Crisis Events: The Roles of Job Crafting and Shared Leadership

**DOI:** 10.1155/2024/3379020

**Published:** 2024-05-16

**Authors:** Yan Wu, Hanqiu Zhu, Wei Tan, Yifeng Liu, Wenjuan Huang

**Affiliations:** ^1^Geriatric Hospital Affiliated to Wuhan University of Science and Technology, Wuhan 430081, China; ^2^School of Management, Wuhan University of Technology, Wuhan 430070, China; ^3^Psychological Depression Ward, Wuhan Mental Health Center, Wuhan 430012, China

## Abstract

**Aims:**

Building on conservation of resources theory, our study investigates how inclusive human resource management (IHRM) promotes nurses' innovative behavior through job crafting and further examines the moderating role of shared leadership.

**Background:**

Nurses' involvement in innovation is essential to improve nursing care delivery and accommodate changing medical environments, especially in the face of crisis events like the COVID-19 outbreak. However, knowledge about the relationship between human resource management and nurses' innovative behavior remains scarce.

**Methods:**

We collected three-wave data from 338 on-duty registered nurses at four public hospitals in China from November 2022 to January 2023. We used SPSS 22 to conduct hierarchical regressions to test our hypotheses.

**Results:**

IHRM positively predicted innovative behavior of nurses with the mediating role of job crafting. In addition, we found that IHRM was more effective in promoting job crafting and subsequent innovative behavior when nurses perceived high levels of shared leadership.

**Conclusion:**

IHRM initiated by the organization and shared leadership style are two collaborative approaches to facilitating nurses' job crafting, thereby responding to the imperative need to foster nurses' innovative behavior. *Implications for Nursing Management*. The present study emphasizes the important roles of IHRM and shared leadership in promoting nurses' job crafting and subsequent innovative behavior, providing theoretical and practical implications for nursing management in the current dynamic and challenging environment.

## 1. Introduction

In today's competitive and technologically advanced medical environment, the survival and growth of healthcare organizations depend on their ability to innovate [1]. Hospital innovation is driven by the knowledge and innovative engagement of nursing staff, who contribute by introducing new ideas, acquiring new knowledge, generating new ideas, improving current processes, and discovering new technologies [2]. During public health emergencies like the COVID-19 outbreak, nursing staff not only face increased risks of infection and heavier workloads but also need to adapt to new working styles and optimize treatments to achieve healthcare goals [3]. Hence, it holds immense theoretical and practical significance to investigate how to develop the potential of frontline nurses in elevating the quality of nursing care to overcome major crises that healthcare organizations may encounter [4].

Innovative behavior refers to the intentional generation, promotion, and implementation of new ideas within a work role, group, or organization to enhance role performance [5]. Highly innovative employees can quickly respond to client needs, generate valuable ideas, and optimize processes, which are essential for improving organizational efficiency and effectiveness [6]. In the nursing field, Zhang et al. [7] conceptualized nurses' innovative behavior as generating new ideas, overcoming challenges and obstacles to realize them, and developing new treatment protocols or policies to restore and promote the health of their patients. Workforce aging, the increasing expectations for high-quality care, and the demand for cost-effectiveness impose greater requirements on healthcare organizations to innovate [8]. In this context, a comprehensive understanding of the drivers behind nurses' innovative behavior is critical for the advancement of healthcare.

Although innovative behavior has provoked extensive attention from scholars in business, education, and project management [2, 9, 10], only a limited number of empirical studies have investigated how to promote nurses' innovative behaviors during crisis events [8]. Furthermore, the majority of these studies has primarily focused on leadership styles, such as servant leadership, humble leadership, and transformational leadership, as antecedents [6, 7, 11], while neglecting the influence of organizational factors, such as human resource management (HRM) practices, on nurses' innovative behavior. However, it is widely acknowledged that innovative behavior is considered discretionary extra-role behavior for most nurses and requires tangible and intangible support from the organization to be sustained (e.g., pay and rewards, an innovative climate, and job autonomy [12]). Numerous studies have shown that HRM practices play a crucial role in enhancing employees' abilities, motivation, and opportunities, thereby influencing their attitudes and behaviors towards innovation [13, 14]. Recognizing the importance of developing relevant theories of HRM practices to encourage innovation in hospitals, Renkema et al. [15] underscored the need for further research in this area. As such, our study contributes to the literature by focusing on the discernible role of inclusive human resource management (IHRM) in promoting innovative behavior among nurses.

During crisis events such as pandemics, frontline nurses face increased psychological and physical demands due to infection control, complex doctor-patient relationships, and occupational hazards during isolation [3, 16]. According to the conservation of resources (CORs) theory, the stressful and threatening environment will pose an intense strain on individuals and consequently lead to potential or actual resource losses, which make individuals typically conserve the limited resources available for innovative behavior [17]. In contrast, a well-resourced organizational context shaped by specific management practices can alleviate individuals' strain and increase their willingness to utilize current resources to engage in innovative activities [18]. As a result, healthcare organizations need to provide nurses with external resources to address stressful demands and reduce the uncertainty of innovation through understanding, respect, and appreciation [19]. IHRM values individuals' differences and unique contributions, treats nurses in a fair and equal way, encourages participative decision-making, and helps nurses adapt to the organization [14]. The support provided by IHRM makes nurses feel that the organization appreciates their competence, invests in their development, and cares about their wellbeing [20], and thus they will proactively conduct innovative behavior in public health emergencies.

The COR theory also suggests that individuals proactively seek to acquire valuable resources through investment to realize the incremental value of the resources [21]. As such, we expect that people are more likely to engage in proactive behavior when they have sufficient resources available to them [17]. Job crafting, which refers to individuals initiating changes in job demands and resources to align with their abilities and needs [22], has been recognized as a proactive behavior that enhances nurses' engagement in innovation [9, 23, 24]. Job crafting is particularly advantageous to healthcare organizations as these skills can be easily acquired and transferred through training and practical experience in an organizational context [25]. IHRM provides nurses with access to more information and resources, increases their participation and sense of control, and allows them to accumulate knowledge and skills [12, 26]. As a result, nurses are more motivated to craft their jobs, which can significantly impact their innovation [18]. Therefore, job crafting may be a crucial mechanism that transforms the resources provided by IHRM into innovative behavior among nurses.

As previously mentioned, nursing leadership is the key to inducing nurses' innovative behavior and transformative change in hospitals [27]. Informal leadership roles serve as the driving force for nurses to generate novel ideas, set new goals, and implement useful techniques [11], and exploring new insights into nursing leadership helps to tackle varied drivers in the multiple contexts of modern healthcare [28]. Our study complements the research by highlighting the interaction between IHRM adopted by the organization and shared leadership among unit nurses in healthcare delivery. Chen et al. [29] denoted that shared leadership is a dynamic process of interaction among members aiming to achieve unit and/or organizational goals, where members work together to set goals, make decisions, learn and mentor, and support and encourage each other [30], and thus, it can be synergistic with IHRM to generate valuable resources for nurses to craft their job and engage in innovate behavior.

**Figure 1:**
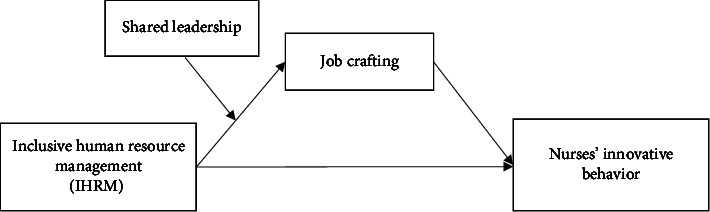
Hypothesized moderated mediation model.

In summary, this study aims to construct a moderated-mediation model (see Figure 1) to examine the internal mechanism and boundary condition of IHRM influencing innovative behavior among nursing staff. Specifically, we seek to answer the following three research questions: (a) whether IHRM can be a critical facilitator of nurses' innovative behavior, (b) whether job crafting can mediate the relationship between IHRM and innovative behavior, and (c) whether shared leadership can amplify the positive effects of IHRM on job crafting and nurses' innovative behavior. We tested our hypotheses through a time-lagged study where questionnaires were administered to registered nurses from four public hospitals in Wuhan between November 2022 and January 2023. The findings from our research can (a) contribute to the literature on the multilateral factors influencing nurses' innovative behavior and the theoretical rationale underlying the relationship between IHRM and nurses' innovation and (b) provide insights for HR practitioners in healthcare to adopt appropriate HRM policies and practices and develop interventions that promote shared leadership and job crafting, thus fostering nurses' innovative behavior.

## 2. Theory and Hypotheses

### 2.1. IHRM and Nurses' Innovative Behavior

IHRM originates from the concept of inclusion and has received widespread attention from scholars over the past two decades. Inclusion, from a leadership perspective, refers to the leader's words and actions that demonstrate an invitation and appreciation for employee contributions [16]. From an involvement perspective, inclusion represents individuals perceiving themselves as part of the organization's key processes [26]. Relationally, inclusion entails building strong relationships with employees and encouraging their participation [31]. From an equity standpoint, inclusion means that employees have the right to express themselves and be appreciated regardless of their social status or class [32]. Taking the optimal distinctiveness perspective, Shore et al. [20] identified inclusion as the extent to which employees feel respected in their work teams based on how well the organization fulfills their need for belongingness and uniqueness. Building on the abovementioned literature, IHRM reflects multiple inclusion-based practices including fair employment policies, participatory decision-making, employee recognition and acceptance, diverse value stimulation and utilization, and rights respect and empowerment [33]. Zhao et al. [14] defined IHRM as a series of interdependent HRM policies, functions, and practices that respect employee differences, recognize employee contributions, accommodate employee mistakes, realize employee strength, encourage employee involvement, and constrain interpersonal conflict to achieve organizational goals. Prior studies identify IHRM as a holistic system consisting of diverse selection (e.g., recruiting heterogeneous employees with multiple knowledge and skills [12]), personalized configuration (e.g., valuing and leveraging employee differential strengths [13]), inclusive development (e.g., providing diversified skills training [34]), participatory assessment (e.g., encouraging participation in the formulation of performance indicators [35]), and targeted compensation (e.g., establishing equal pay system, decent salaries, and good benefits [26]). IHRM ascertains that employees are appreciated and rewarded for their contributions and treated equitably across different position levels [16], and scholars have proven its plausibility in relation to employee creativity [14], wellbeing [12], and job satisfaction [35] within the organization.

Nurses' innovative behavior refers to the process of changing routines or employing new methods and technologies to optimize workflow and enhance patient satisfaction with healthcare delivery [1]. The International Council of Nursing (ICN) recognizes that nursing innovation encompasses the following three phases: idea generation, idea promotion, and idea implementation, and emphasizes its important role in promoting wellness, preventing disease, and improving the quality of healthcare [6]. Innovation is a complex and prolonged process that continuously consumes nurses' psychological, physical, and knowledge resources [4]. During crisis events, healthcare workers experience immense pressure to maintain emotional composure while dedicating themselves to patient care, which often leads to mental dissonance and continued loss of resources [3, 16]. The COR theory postulates that people are motivated to protect their current resources and acquire new resources to defend themselves against future resource loss [18]. As a result, nurses may reduce high-risk innovative behavior if they are aware of potential or actual resource loss [17]. Therefore, it is important to provide nurses with sufficient resources to supplement what has been depleted to promote their innovative behavior [21]. Previous research has confirmed that the workplace represents a proximate environment in which employee resource pools and associated work outcomes are affected by HRM practices [12]. In this vein, IHRM can be viewed as a source of resources for nurses to cope with highly demanding work, which can effectively compensate for nurses' loss of resources and enable them to possess sufficient psychological resources to innovate during crisis events [20].

Specifically, the diverse selection of IHRM emphasizes the need for hospitals to construct multifaceted talent teams, with nurses' gender, age, education, and experience structure displaying a certain degree of complementarity [36]. Nursing staff with various experiences and backgrounds are more likely to form differentiated knowledge structures and critical thinking patterns. These heterogeneous cognitive resources are conducive to the formation of nurses' open-minded visions, which in turn inspire them to develop and implement novel and useful ideas to improve service methods that benefit the patients [33]. Meanwhile, inclusive development focuses on individualized training for nurses to provide them with knowledge and skill resources [35], such as professional knowledge, clinical skills, technology proficiency, emergency response, and communication skills, which are critically needed to cope with creative requirements. Participatory assessment empowers nurses to engage in the decision-making process and tailor performance appraisal approaches based on specific job characteristics. This practice ensures that nurses feel their viewpoints are respected and have access to organizational information resources, thus significantly enhancing their motivation to conduct innovative behaviors [31]. In addition, IHRM advocates the application of flexible work systems such as shift changes, rotations, and leave transfers in the task allocation process and gives nurses a sense of autonomy through empowerment practices [13]. Job flexibility and autonomy provided by personalized configuration allow nurses to proactively engage in innovative behavior such as creative problem solving to realize resource augmentation [37]. Finally, one's commitment to risky creative activities requires the necessary incentives and support, and favorable compensation and benefits can effectively hedge the risk of loss associated with innovation [26]. Targeted compensation adjusts compensation levels, structures, and strategies in response to feedback from nursing staff to ensure that each nurse's contribution can be valued and rewarded. Sufficient material resources constitute a critical prerequisite for promoting nurses' readiness and willingness to innovate [38]. In summary, IHRM practices serve as valuable resources invested by healthcare organizations to increase nurses' resource stock (e.g., enhancement of professional skills, increase in reward, and reduction of role ambiguity [12]) through diverse selection, inclusive development, personalized configuration, participatory assessment, and favorable compensation. As such, nurses with adequate resources (e.g., support from the hospital, training, and development opportunities) will generate more ideas about public health and nursing service and put them into practice [17]. Thus, we hypothesize the following.

**Hypothesis 1 .**

IHRM positively affects nurses' innovative behavior.

### 2.2. The Mediating Role of Job Crafting

Job crafting is a bottom-up approach to autonomous job redesign that diverges from the top-down approach typically implemented by organizations and places emphasis on employees' initiative to make changes in their work [39]. Following the job demands-resources model, research has identified the following four types of job crafting [22]: increasing structural job resources (diverse resources and autonomy to help employees achieve their goals), increasing social job resources (social support and feedback from managers, colleagues, and others), increasing challenging job demands (accomplish more difficult tasks to improve knowledge and skills), and decreasing hindering job demands (mitigate resource depletion when faced with excessive demands). Nurses who engage in job crafting proactively align their talents, strengths, and interests with their work environment, enabling them to achieve a balance between job demands and resources [23, 25].

We draw on the COR theory to scrutinize how IHRM influences nurses' innovative behavior by fostering their job crafting. The COR theory highlights human's evolutionary need to conserve and acquire resources for survival that is central to the genetics of human behavior [17]. A fundamental principle of the COR theory relates to the notion of resource investment, that is, when people possess sufficient resources, they tend to proactively invest resources in behaviors designed to compensate for prior resource losses or to acquire additional resources [18]. Consistent with this postulation, employees tend to engage in job crafting to invest their resources to create a better work environment when a resource surplus occurs [21]. As earlier mentioned, IHRM implemented by the healthcare institution creates a resourceful organizational context for nursing staff [12]. We, therefore, expect that job crafting can be regarded as an agentic behavior of resource investment that provides individuals with beneficial ways to utilize and transform useful resources derived from IHRM to achieve a positive resource gain spiral [18], such as generating energy, self-efficacy, interpersonal capital, and diverse skills that make employees more resilient to resource loss and more willing to undertake the risks of innovative behavior [40].

We first argue that IHRM can stimulate job crafting among nurses by supplying them with sufficient job resources [39]. IHRM allows nurses to exert considerable influence over organizational decisions and gives them access to more information and resources [14], which largely increases nurses' sense of control and further facilitates their job crafting. Furthermore, training and rotations provided by IHRM enable nurses to accumulate knowledge, skills, and abilities to improve their occupational competence and job performance [34]. Research has demonstrated that individuals with higher occupational competence are more likely to craft their job in a more skillful and professional manner [41]. Nurses who have occupational competence as an available resource can feel more comfortable crafting the way they work. More importantly, Van Wingerden and Poell [42] have emphasized that the opportunity for job crafting is an indispensable factor in influencing individuals' decisions to engage in this behavior. IHRM makes nurses perceive more opportunities to alter their job by providing greater job autonomy. For instance, organizations grant nurses specific job authorizations and autonomy in their work arrangements, allowing them to choose appropriate training, performance evaluation approaches, and benefits tailored to individualized needs. Taken together, we propose that nurses perceiving high IHRM will proactively engage in job crafting based on their talents, interests, and strengths [23]. Thus, we hypothesize the following.

**Hypothesis 2 .**

IHRM is positively related to nurses' job crafting.

We further predict that job crafting can positively influence nurses' innovative behavior because job crafting is a proactive behavior that provides nurses with opportunities to access useful resources that promote work goal attainment while reducing obstructive job demands that deplete physical and psychological resources [19]. Specifically, structural job resources such as increased discretion in work enable nurses to decide how to perform tasks independently and provide them with more chances to try different techniques, methods, and procedures to solve complex medical problems [40], which improves nurses' feeling of obligation to innovate [4]. Social job resources such as head nurses' support can help nurses acquire and accumulate emotional resources that reduce the innovation risk and prevent nurses from falling into the loss spiral due to resource shortage [43]. In line with the COR theory, nurses who possess plentiful material and emotional resources face less pressure at work and are more inclined to exhibit innovative behaviors to enhance self-worth [18, 42]. Meanwhile, increasing challenging job demands can significantly promote job vitality, which to some extent enhances individuals' cognitive flexibility and improves nurses' creative thinking during crisis events [44]. Hindering job demands that impede nurses' growth and development, such as role conflict and bureaucracy, may prevent the optimal functioning of tasks, bring unnecessary stress, and lead to negative emotions in nursing staff [25]. When individuals experience a loss of resources at work, they will adopt a defensive attitude towards behaviors that consume resources [21]. Job crafting avoids resource loss by reducing obstructive job demands, further promoting innovative behaviors among nurses. Taken together, job crafting can promote nurses' innovative behavior by simultaneously facilitating a “resource gain path” that increases job resources and challenging job demands, and inhibiting a “resource loss path” that generates obstructive job demands [19, 22]. Thus, we hypothesize the following.

**Hypothesis 3 .**

Job crafting is positively related to nurses' innovative behavior.

Based on the arguments above, we expect that IHRM provides a platform for nursing staff to acquire resources that enhance their abilities, motivation, and opportunities for job crafting, which in turn promotes their innovative behavior. Thus, we hypothesize the following.

**Hypothesis 4 .**

Job crafting mediates the positive relationship between IHRM and nurses' innovative behavior.

### 2.3. Shared Leadership as a Boundary Condition

Shared leadership, as an emerging leadership style, refers to a group dynamic interaction process whereby different members are selected to assume leadership roles at different stages according to changes in the external environment and group members' strengths to achieve the common goals of the group [45]. Leadership style directly affects the nurses' perception and interpretation of HRM practices and further influences their attitudes and behaviors [46]. Shared leadership delivers consistent signals with IHRM that team members are encouraged to participate in decision-making and extensive information sharing [47], making nurses feel a sense of autonomy and initiative to utilize the resources from IHRM for job crafting and innovative behavior [48].

In this sense, we expect that shared leadership may reinforce the positive relationship between IHRM and job crafting. Research has identified leadership style as the contextual force that enables or constrains the eventual implementation of job crafting [27]. Shared leadership dynamically shifts leadership responsibilities among group members based on their expertise and competencies in relation to specific task situations [29]. It allows nurses to break away from the constraints of their previous roles and routines so that they can try out various work methods, fully develop their potential, and improve their efficacy [45]. Thus, nurses under high shared leadership have a higher sense of competence and believe that they can better utilize the opportunities provided by IHRM to craft their job [32]. In addition, since job crafting is the readjustment and optimization of nurses' tasks and relationships related to their work, this process involves the change of the original work pattern and approach, which may be impeded and constrained by their colleagues [49]. Shared leadership breaks down the traditional interpersonal interaction pattern among group members [30], and nurses engage in frequent exchanges and interactions with colleagues to form a sharing, reciprocal, and harmonious relationship [28]. Under high shared leadership, nurses seldom encounter obstacles and constraints from their colleagues and tend to utilize the resources provided by IHRM to conduct job crafting [47]. In contrast, although IHRM enhances nurses' abilities, motivation, and opportunities for job crafting, nurses under low shared leadership lack a sense of competence and are hindered by colleagues, which prevent them from job crafting. Thus, we hypothesize the following.

**Hypothesis 5 .**

Shared leadership moderates the relationship between IHRM and job crafting such that this positive relationship is stronger when shared leadership is high (vs. low).

As previously mentioned, shared leadership accentuates the positive impact of IHRM on nurses' job crafting through power and responsibility sharing, team learning, member support, and mutual skill development, and job crafting provides nurses with more resources to engage in innovative behavior [24]. In contrast, nurses who perceive low shared leadership are less likely to utilize the resources derived from IHRM to craft their job and display innovative behavior. Thus, we hypothesize the following

**Hypothesis 6 .**

Shared leadership moderates the indirect effects of IHRM on nurses' innovative behavior via job crafting such that this indirect effect is stronger when shared leadership is high (vs. low).

## 3. Method

### 3.1. Sample and Procedure

We collected data from on-duty registered nurses at four public hospitals in China. Researchers contacted the cochiefs of nursing staff at four public hospitals in Wuhan that are top in healthcare services and medical technology and explained the purpose and procedures of the study to obtain permission. With the assistance of head nurses, a disclosure statement was delivered to the nurses who participated in the questionnaire survey, including the academic purpose of our research, the principles of voluntariness, anonymity, and confidentiality, which would constitute the informed consent of nurses to participate in the investigation. Nurses participating in the study should meet the following criteria: (a) above 18 years of age; (b) hold a Chinese registered nurse license; and (c) work full time. We excluded nursing staff not directly involved in patient care and who were on leave, such as maternity, sick, and vacation leave. Our research adopted a time-lagged design to collect data at 3 time points with a 1-month interval to minimize the influence of common method variance [50]. Participants responded to the questionnaire items measuring the predictors (IHRM and shared leadership), mediator (job crafting), and outcome variable (innovative behavior) at time points 1, 2, and 3, respectively, and the self-generated anonymous codes were used to guarantee the effective match of the three-wave survey.

Data were collected from November 2022 to January 2023 during the reopening of the epidemic.  Table 1 presents the data collection procedures. In light of the population mobility constraints imposed by the epidemic in China, we utilized online methods to collect data and emailed a link with the questionnaire to participants. At Time 1, we sent questionnaires to 400 nurses at four hospitals and asked about their demographics, perceived IHRM, and perceived shared leadership. We received 374 responses. At Time 2, nurses who had completed the Time 1 survey measured job crafting. 352 valid questionnaires were returned. At Time 3, the remaining 352 nurses reported their innovative behavior. We received 345 completed questionnaires. After eliminating 7 invalid questionnaires due to missing and unmatched data, we ultimately obtained a valid sample of 338 nurses, yielding a response rate of 84.5%.  Table 2 presents the participants' demographic characteristics. Among the participants, 22.2% were male and 77.8% were female. 18.3% were 25 years old or below, 49.1% were in the 26–35 age range, 29.0% were in the 36–45 age range, and the remaining 3.6% were 46 years old or above. 37.6% reported an associate's degree or below, 53.3% had a bachelor's degree, and the remaining 9.1% had a master's degree or above. 29.9% had 2 years of experience or below, 25.7% had 3–5 years of experience, 27.8% had 6-7 years of experience, and 16.6% had 8 years of experience or above. 47.3% were nurses, 38.5% were senior nurses, and 14.2% were supervisor nurse or above.

### 3.2. Measures

All measures utilized in this study were drawn from prior research and have been demonstrated to possess satisfactory reliability and validity over time. A 5-point Likert scale was adopted to measure all items, ranging from 1 (strongly disagree) to 5 (strongly agree). The definitions and measures of the variables are presented in Table 3.

### 3.3. IHRM

IHRM was measured using 20 items adapted from Zhao et al. [14] in the Chinese context. This scale included the following five dimensions: diverse selection (five items, e.g., “in my organization, there is no discrimination against gender, age, ethnicity, religion, nationality, and dialect during recruitment and selection”), personalized configuration (five items, e.g., “My organization considers job demands, nurses' needs, and strengths during task assignment”), inclusive development (five items, e.g., “my organization provides differentiated and individualized training programs to meet the various needs of nurses”), participatory assessment (five items, e.g., “in my organization, performance appraisal emphasizes results feedback, problem diagnosis, and recommendation solicitation”), and targeted compensation (five items, e.g., “compared with similar positions in other hospitals, the compensation offered by my organization is fair and reasonable”). Cronbach's *α* was 0.930.

### 3.4. Shared Leadership

Shared leadership was assessed using 16 items from Hoch and Kozlowski [30]. This scale consisted of the following three dimensions: team learning (four items, e.g., “our team actively searches our own performance for deficits”), perceived team support (five items, e.g., “my team really cares about my wellbeing”), and member-member exchange (seven items, e.g., “my team understands my problems and needs”). Cronbach's *α* was 0.941.

### 3.5. Job Crafting

We measured job crafting using 21 items from Cheng et al. [25], which had been validated among nurses in public hospitals in China. The scale consisted of the following four dimensions: increasing structural job resources (five items, e.g., “I make sure that I use my capacities to the fullest”), increasing social job resources (five items, e.g., “I ask others for feedback on my job performance”), increasing challenging job demands (five items, e.g., “If there are new developments, I am one of the first to learn about them and try them out”), and decreasing hindering job demands (six items, e.g., “I make sure that my work is mentally less intense”). Cronbach's *α* was 0.848.

### 3.6. Innovative Behavior

We measured nurses' innovative behavior using a 6-item scale adapted from Scott and Bruce [5]. A sample item was “in my work, I will actively seek new methods, techniques, and procedures.” Cronbach's *α* was 0.928.

### 3.7. Control Variables

Following previous research on innovative behavior [10, 11], control variables of nurses' gender, age, education, and experience were included.

### 3.8. Analytical Approach

We used SPSS 22 to conduct hierarchical regressions to test our hypotheses. Specifically, we constructed regression models, respectively, with job crafting and nurses' innovative behavior as dependent variables to estimate the standardized regression coefficients between main variables. To test the moderating effects, we created an interaction term by mean centering IHRM and shared leadership and performed the simple slope tests following Aiken and West's [51] procedures. Finally, we conducted bootstrap analyses to generate 95% bias-corrected confidence intervals (CIs) for mediation and moderated mediation effects using Hayes' [52] PROCESS macro. The moderated mediation effect was significant if 95% CI of difference between the indirect effects under high (+1 SD) and low (−1 SD) levels of the moderator excluded 0.

## 4. Results

### 4.1. Confirmatory Factor Analyses (CFA)

We compared measurement models through a series of CFAs using Mplus 8.0. As shown in Table 4, the expected four-factor model yielded a good fit to the data (*χ*^2^(98) = 269.189, CFI = 0.949, TLI = 0.937, RMSEA = 0.072, and SRMR = 0.031) and the hypothesized model fit the data better than the alternative models with fewer factors, indicating that there was an acceptable discriminant validity between the focal variables.

### 4.2. Common Method Variance (CMV)

Following Podsakoff et al.'s [50] recommendations, we first performed Harman's single-factor test to examine the CMV. The results of exploratory factor analysis (EFA) indicated that the first common factor only accounted for 27.1% of the variance, which was less than the threshold of 40% and less than half of the 69.2% of the total variance explained. In addition, results of the CFA showed that the goodness of fit of the five-factor model with an additional latent common method factor was not improved significantly (Δ*χ*^2^/*df* = 0.127, ΔCFI = 0.011, ΔTLI = 0.013, ΔRMSEA = 0.008, and ΔSRMR = 0.007) than the expected four-factor model, revealing that the CMV of our study was no serious.

### 4.3. Descriptive Statistics and Correlations

The mean scores for IHRM, shared leadership, job crafting, and innovative behavior were 3.351 ± 0.718, 3.613 ± 0.716, 3.766 ± 0.393, and 3.753 ± 0.697, respectively. IHRM was positively correlated with nurses' innovative behavior (*r* = 0.261, *p* < 0.001) and job crafting (*r* = 0.254, *p* < 0.001). Furthermore, job crafting showed a significant positive correlation with nurses' innovative behavior (*r* = 0.369, *p* < 0.001), providing preliminary support for the hypotheses.

### 4.4. Hypothesis Testing

**Figure 2:**
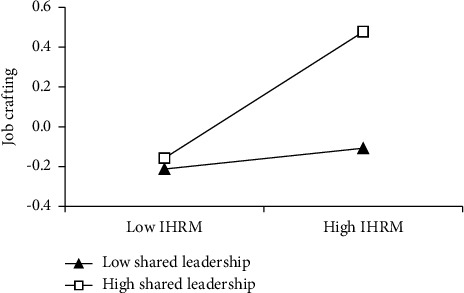
The moderating effect of shared leadership on the relationship between IHRM and job crafting.

**Figure 3:**
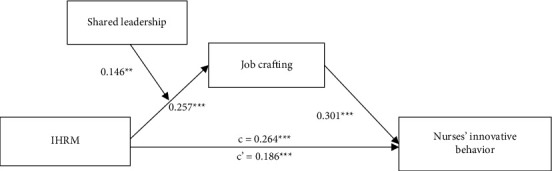
Hypothesized model with standardized regression coefficients. *Note*. *N* = 338. ^*∗∗*^*p* < 0.01, ^*∗∗∗*^*p* < 0.001.

The results of hierarchical regression analyses are presented in Table 5. After controlling for nurses' gender, age, education, and experience, the coefficient of IHRM on nurses' innovative behavior was significant and positive (*β* = 0.264, *p* < 0.001; model 3), supporting Hypothesis 1. In support of Hypotheses 2 and 3, the results revealed that IHRM positively affected job crafting (*β* = 0.257, *p* < 0.001; model 1) and job crafting had a positive impact on nurses' innovative behavior (*β* = 0.350, *p* < 0.001; model 4). As shown in Table 5, after adding job crafting, the positive coefficient between IHRM and nurses' innovative behavior decreased from (*β* = 0.264, *p* < 0.001; model 3) to (*β* = 0.186, *p* < 0.001; model 5); however, job crafting was still positively related to nurses' innovative behavior (*β* = 0.301, *p* < 0.001; model 5). Thus, we concluded that job crafting partially mediated the impact of IHRM on nurses' innovative behavior. In addition, we used PROCESS macro to examine the statistical significance of the mediation effect. Bootstrap results revealed that the indirect effect of IHRM on nurses' innovative behavior via job crafting was significant (indirect effect = 0.078, 95% CI = [0.039, 0.121]), providing additional support for Hypothesis 4. As shown in Table 5, the interaction between IHRM and shared leadership positively affected job crafting (*β* = 0.146, *p* < 0.01; model 2). We further conducted the simple slope tests and plotted the moderating effect in Figure 2. The results showed that the link between IHRM and job crafting was significant and positive under high levels of shared leadership (*β* = 0.158, *p* < 0.001) but nonsignificant under low levels of shared leadership (*β* = 0.044, *p*=0.303), supporting Hypothesis 5. Finally, we used the bootstrap approach to calculate the conditional indirect effect to test Hypothesis 6. The indirect effect of IHRM on nurses' innovative behavior through job crafting was significantly positive when shared leadership was high (indirect effect = 0.078, 95% CI = [0.027, 0.145]) but nonsignificant when shared leadership was low (indirect effect = 0.022, 95% CI = [−0.040, 0.079]), and the difference between the indirect effects was significant (difference = 0.057, 95% CI = [0.003, 0.128]), supporting Hypothesis 6. The hypothesized model and standardized regression coefficients are presented in Figure 3.

## 5. Discussion

Our research answered recent calls to shed light on multiple factors that affect nursing staff's innovative behavior [8, 15]. We conducted a time-lagged field study to investigate whether, how, and when IHRM could promote nurses' innovative behavior by focusing on the mediating role of job crafting and the moderating effect of shared leadership. The results demonstrated that nurses perceiving higher levels of IHRM are more likely to engage in job crafting and consequently display more innovative behavior to improve current healthcare processes, services, and products during crisis events and this indirect relationship strengthens when nurses perceive higher shared leadership. Specifically, we obtained the following three findings which contribute to the existing HRM-innovation relationship literature.

### 5.1. Theoretical Implications

#### 5.1.1. IHRM Is Positively Associated with Nurses' Innovative Behavior

People's interpretations of inclusion and related practices may vary across cultures. In the Western context, scholars have mainly concerned with the fair and equal treatment of diverse employees, such as providing employees with opportunities to participate in organizational processes and decision-making through practices such as information sharing and employee involvement, thereby reducing discrimination and conflict inside and outside the organization [32, 36, 37]. However, inclusion in the Chinese culture (also known as *bao rong*) has a broader meaning that consists of two critical elements, “seeking commonalities” and “utilizing differences,” highlighting the importance of making employees feel respected and integrated into the organization to experience a sense of belonging while preserving and utilizing employee uniqueness to optimize their personal strengths [26]. Therefore, IHRM may be more connected to employee creativity and innovation in the Chinese context [33]. Our results revealed a positive relationship between inclusive management practices and nurses' innovative behavior, which is aligned with the results of similar research conducted in business enterprises [14]. As such, our study extends previous literature by substantiating the applicability of IHRM to nursing staff in the healthcare field.

However, the investigated hospitals in this study only achieved a medium level of IHRM (3.351 ± 0.718), which is lower than the findings of Zhao et al. [12] who examined knowledge-intensive companies. This suggests that healthcare organizations may lag behind in the development and implementation of HRM practices compared to business firms [8]. Considering the pressure, increasing need for innovation, and limited resources faced by nurses during a crisis, it is crucial for healthcare organizations to be more proactive in implementing IHRM to provide nurses with valuable physical, emotional, knowledge, and skill resources that support innovation [16, 18]. This study provides a theoretical foundation for these organizations to understand and develop relevant HRM policies that promote nurses' innovative behavior.

In addition, although a significant amount of research has focused on appropriate leadership styles as antecedents of nurses' innovative behavior, such as servant leadership, humble leadership, and transformational leadership [6, 7, 11], studies examining how organizational factors such as HRM policies and practices impact nurses' innovative behavior remain scarce. Accordingly, our research empirically confirms the crucial role of IHRM in facilitating innovative behavior, expanding studies on the antecedents of nurses' innovation and responding to the call from Renkema et al. [15] to explore nursing-related HRM theories to encourage innovation beyond leadership.

#### 5.1.2. Job Crafting Mediates the Relationship between IHRM and Nurses' Innovative Behavior

The current study investigated the mediating process that transforms the beneficial impacts of IHRM into nurses' innovative behavior, and notably, the empirical evidence pertinent to this topic has primarily focused on employees' positive psychological states, such as psychological safety and psychological empowerment [8, 10]. Our findings help add to the current knowledge by demonstrating the mediating role of job crafting between IHRM and nurses' innovative behavior. Cheng et al. [25] reported that job crafting is increasingly recognized as an important concept in the nursing field, with its application expanding across different countries and it plays an essential role in improving nurses' person-environment congruence and wellbeing, especially during crisis events. Ghazzawi et al. [23] examined the role of personality in the Arab World in predicting job crafting and confirmed the positive influence of job crafting on subjective wellbeing of nurses. However, there exists a knowledge gap regarding the relationship between HRM practices, job crafting, and innovative behavior among nurses. The resource investment principle of the COR theory provides a perspective for understanding this issue, which posits that people will proactively invest resources already acquired in the hope of obtaining more resources to improve the environment [18]. In line with Van Wingerden and Poell [42], our findings revealed that IHRM can establish a resource-rich working condition through diverse selection, inclusive training, personalized configuration, participatory assessment, and targeted compensation [14], and this, in turn, motivates nurses to engage in job crafting as a form of resource investment [17]. Our results further suggested that additional increased resources and decreased job demands associated with job crafting enable nurses to devote more time and energy to innovative activities that enhance existing healthcare processes, products, and services [22], thus echoing the research of Khan et al. [24]. In this way, our study unveils the theoretical foundations between IHRM and innovation in the nursing context.

#### 5.1.3. Shared Leadership Moderates the Positive Effects of IHRM on Nurses' Job Crafting and Subsequent Innovative Behavior

As a positive leadership style, our findings shed light on the important role of shared leadership in reinforcing the effectiveness of IHRM on nurses' job crafting and innovative behavior, affirming past research that indicated the synergistic effects of positive leadership styles and HRM practices in response to crisis events [10, 46]. Prior studies have highlighted the advantages of vertical leadership, such as transformational leadership, in fostering innovative behavior among nurses during a pandemic [7], neglecting how horizontal models of shared leadership function. Indeed, despite the tremendous pressure during the crisis, nurses have demonstrated a willingness to craft their job to effectively change how they work and interact with patients to improve the quality of nursing service [4], and this process may be influenced by the initiative and autonomy of nursing staff [8]. As an informal collective leadership pattern characterized by members' proactive involvement, self-management, and mutual leading, shared leadership emphasizes the sharing of leadership roles among members, such as decision-making, power, and responsibilities [45]. In line with this notion, our results indicated that shared leadership can function as a facilitator that grants nurses greater autonomy and initiative to utilize resources from IHRM to engage in job crafting and innovative behavior [28]. This study not only extends the current understanding of the boundary condition of IHRM but also confirms the reasoning that leadership has the capacity to promote HRM process [48]. Therefore, healthcare organizations should strive to enhance the levels of shared leadership to maximize the benefits of IHRM.

### 5.2. Implications for Nursing Management

Our research provides several practical implications for HR practitioners and nursing leaders in healthcare organizations. Nurses' innovative behaviors have emerged as a critical issue in recent healthcare research [6] and have been proven to have a positive impact on improvements in the quality and effectiveness of nursing services during a pandemic [7]. To create a supportive environment for innovation among nursing staff, organizations should predominantly concentrate on nurses' vulnerability to stress during a crisis event and provide them with adequate tangible and intangible resources [2, 3]. First, HR practitioners are expected to stimulate nurses' abilities, motivations, and opportunities to innovate by adopting various inclusive practices to increase nurses' stock of resources, reduce their pressure, and leverage their strengths [12]. These HRM policies and practices should include fair employment, flexible work, value appreciation, diverse training, participatory decision-making, decent compensation, and targeted rewards [14]. More importantly, HR practitioners in healthcare should also actively involve nursing staff in these available practices and capitalize on nurses' feedback and opinions to timely adjust HRM practices for their potential innovation benefits [15].

Second, we can understand from this research that job crafting is an essential agentic behavior that has a discernible influence on innovative behavior. Given an array of uncertainties associated with the pandemic, nurses should actively redesign their work to achieve a balance between job demands and resources [19]. Accordingly, nursing managers should create a favorable environment for nurses' job crafting, such as creating autonomous work conditions and avoiding unnecessary interference and monitoring of nurses [25]. By proactively seeking opportunities to craft their job according to talents, interests, and strengths, nurses can acquire valuable resources needed for innovation.

Third, our research revealed that IHRM is more likely to facilitate nurses' job crafting and innovative behavior when nurses perceive higher levels of shared leadership. In the changing medical environment, the complexity of tasks and the urgency of time make it difficult for the traditional vertical leadership style to accomplish work tasks with high-quality [27]. To better adapt to environmental changes, nursing leaders in healthcare organizations should improve the levels of shared leadership. Specifically, team members should be encouraged to share leadership authority and responsibilities to promote the effective achievement of collective goals [29]. In addition, team members need to learn from each other and try new methods to improve their performance.

### 5.3. Limitations

Our research has several limitations. First, IHRM, shared leadership, job crafting, and nurses' innovative behavior were all self-reported by the nurses, which would inevitably raise concerns about subjective cognitive bias and social desirability. Although we adopted a time-lagged design to measure the main variables at three-time points during questionnaire distribution to mitigate the CMV and Harman's single-factor test and latent common method factor approach illustrated that this problem was not serious, a single source of data could still have some limitations [50]. Second, this study was conducted in four public hospitals in China. The monocultural and organization-specific context may limit the generalizability and extensibility of our findings. However, the current data do not allow for a direct empirical examination of how contextual factors (e.g., culture, organization, and healthcare systems) might influence the relationships between IHRM and related variables through a comparative design [23]. Third, researchers have demonstrated that time-separated measures can be effective in reducing the potential limitations of cross-sectional data (e.g., CMV and reverse causality [15]). However, the one-month time interval recommended by Podsakoff et al. [50] is insufficient to validate the long-term impact of IHRM on nurses' innovative behavior, which may change as the crisis event continues to progress [16].

### 5.4. Future Research

We also provide some avenues for future research. First, we investigated the impact of IHRM on nurses' innovative behaviors using individual-level data because prior research indicated that individuals might make differential interpretations of organizational intentions behind HRM practices [12]. However, individuals perceived IHRM could hardly demonstrate the objectivity of HRM practices. Therefore, future research could collect data at both organizational- and individual-level to examine how organization-implemented IHRM can influence nurses' innovation through multilevel analysis. Second, considering the differences between Eastern and Western cultures, individuals may have different understandings of “inclusion” and China's unique culture (e.g., high collectivism) makes people more eager to belong or merge into the group, which may lead to a greater positive impact of IHRM on related outcomes in Chinese culture. Accordingly, we encourage future research to explore the role that cultural factors play in employees' perceptions of IHRM and perform a cross-cultural comparative analysis to generalize our findings. In addition, future research could extend our findings to other healthcare organizations since the innovative requirements and expectations are different for nursing staff under different healthcare systems [7]. Third, it is a feasible direction for future research to expand the present study through longitudinal design to compare the impacts of IHRM on innovative behavior during a crisis event like a pandemic with the impacts under ordinary situations. In this way, scholars could continue to explore whether IHRM and job crafting can predict changes in nurses' innovative behavior over time. Finally, future studies could examine more extensive mediating variables (e.g., psychological capital, thriving at work, and gratitude) to expand potential theoretical explanations between IHRM and innovative behavior [9]. We also recommend future research to explore other boundary conditions such as proactive personality and servant leadership to extend our findings [2].

## 6. Conclusion

Promoting nurses' innovative behavior can be an effective way for hospitals to acquire sustainable competitive advantage in today's VUCA (i.e., volatile, uncertain, complex, and ambiguous) medical environments. Based on the COR theory, the current study constructs a moderated-mediation model to explore whether, how, and when IHRM can motivate nurses to be involved in innovative work. We tested our theoretical model through a time-lagged field study and the results showed that IHRM is positively related to job crafting, which in turn promotes nurses' innovative behavior. In addition, we uncover when IHRM has a more pronounced influence on nurses' job crafting and innovative behavior by selecting shared leadership as a boundary condition. We found that the direct linkage between IHRM and job crafting and the indirect effect of IHRM on nurses' innovative behavior through job crafting is significant and positive only when the levels of shared leadership are high rather than low. To conclude, our research indicates that IHRM and shared leadership style are two collaborative approaches to facilitating nurses' job crafting and subsequent innovative behavior during crisis events. As such, hospitals and nursing facilities may improve the level of job crafting by adopting IHRM policies and practices that value the personalized values and demands of nurses and enhancing perceived shared leadership that underscores collective learning, mutual support, and member-member exchange, thereby fostering the innovation of nurses.

## Figures and Tables

**Table 1:** Data collection procedures.

Research design	Time points	Data collection period	Evaluator	Variables	Questionnaires distributed	Valid questionnaires returned
Time-lagged questionnaire survey	Time 1	1 November 2022–3 November 2022	Nurse self-report	Demographics, IHRM, and shared leadership	400	374
	Time 2	1 December 2022–3 December 2022	Nurse self-report	Job crafting	374	352
	Time 3	1 January 2023–3 January 2023	Nurse self-report	Nurses' innovative behavior	352	338

**Table 2:** Demographic characteristics of the participants.

Variable	*N*	%
Gender		
Female	263	77.81
Male	75	22.19
Age (years)		
≤25	62	18.34
26–35	166	49.11
36–45	98	29.00
≥46	12	3.55
Education		
Associate's degree or below	127	37.57
Bachelor's degree	180	53.26
Master's degree or above	31	9.17
Experience (years)		
≤2	101	29.88
3–5	87	25.74
6-7	94	27.81
≥8	56	16.57
Position		
Nurses	160	47.34
Senior nurses	130	38.46
Supervisor nurse or above	48	14.20

**Table 3:** The definitions and measures of study variables.

Variable	Definition	Dimensions	No. of items	Source
IHRM	IHRM refers to a set of interdependent HRM policies and practices that respect employee differences, recognize employee values, leverage employee expertise, enhance organizational equity, and provide employees with autonomy, flexibility, and the necessary support	Diverse selection, personalized configuration, inclusive development, participatory assessment, and targeted compensation	20	[12, 14]
Shared leadership	Shared leadership depicts an interactive process characterized by collaborative decision-making and shared responsibility whereby group members lead each other to achieve goals	Team learning, perceived team support, and member-member exchange	16	[30]
Job crafting	Job crafting is defined as the changes made by employees to balance their job demands and job resources with personal abilities and needs in response to organizational change	Increasing structural job resources, increasing social job resources, increasing challenging job demands, and decreasing hindering job demands	21	[22, 25]
Nurses' innovative behavior	Innovative behavior refers to the intentional generation, promotion, and implementation of new ideas within a work role, group, or organization. Nurses' innovative behavior may manifest as incremental adjustments to current healthcare processes, services, and products or as innovative pragmatic solutions to restore and enhance patients' health	One dimension	6	[5, 7]

**Table 4:** Comparison of measurement models for study variables.

Models	Descriptions	*χ* ^2^	*df*	*χ* ^2^/*df*	Compare model differences	CFI	TLI	RMSEA	SRMR
Four-factor model	IHRM, SL, JC, IB	269.189	98	2.747		0.949	0.937	0.072	0.031
Three-factor model	IHRM, SL, JC + IB	439.090	101	4.347	Δ*χ*^2^(3) = 169.901, *p* < 0.001	0.899	0.880	0.100	0.063
Two-factor model	IHRM, SL + JC + IB	967.325	103	9.392	Δ*χ*^2^(5) = 698.136, *p* < 0.001	0.741	0.699	0.158	0.162
One-factor model	IHRM + SL + JC + IB	1868.844	104	17.970	Δ*χ*^2^(6) = 1599.655, *p* < 0.001	0.472	0.391	0.224	0.189

*Note*. *N* = 338. IHRM = inclusive human resource management, SL = shared leadership, JC = job crafting, IB = nurses' innovative behavior. CFI = comparative fit index, TLI = Tucker–Lewis index, RMSEA = root mean square error of approximation, SRMR = standardized root mean square residual.

**Table 5:** Results of regression analyses.

Variables	Job crafting		Nurses' innovative behavior		
	Model 1	Model 2	Model 3	Model 4	Model 5
Control variables					
Gender	0.023	0.034	0.085	0.080	0.078
Age	−0.071	−0.065	−0.030	−0.024	−0.008
Education	−0.034	−0.036	−0.050	−0.039	−0.040
Experience	0.225^*∗∗*^	0.210^*∗∗*^	0.172^*∗*^	0.097	0.104
Independent variable					
IHRM	0.257^*∗∗∗*^	0.185^*∗∗*^	0.264^*∗∗∗*^		0.186^*∗∗∗*^
Mediator					
Job crafting				0.350^*∗∗∗*^	0.301^*∗∗∗*^
Moderator					
Shared leadership		0.160^*∗*^			
Interaction					
IHRM × shared leadership		0.146^*∗∗*^			
*R*^2^	0.099	0.127	0.101	0.151	0.183
Δ*R*^2^	0.099^*∗∗∗*^	0.028^*∗∗*^	0.101^*∗∗∗*^	0.151^*∗∗∗*^	0.082^*∗∗∗*^
*F*	7.324^*∗∗∗*^	6.849^*∗∗∗*^	7.486^*∗∗∗*^	11.815^*∗∗∗*^	12.360^*∗∗∗*^

*Note*. *N* = 338. ^*∗*^*p* < 0.05, ^*∗∗*^*p* < 0.01, ^*∗∗∗*^*p* < 0.001.

## Data Availability

The data used to support the findings of this study are available from the corresponding author upon reasonable request.
